# Recurred pantoprazole anaphylaxis: a rare case report from Nepal

**DOI:** 10.1097/MS9.0000000000003863

**Published:** 2025-09-15

**Authors:** Aseem Wagle, Pradeep Thapa, Kiran Lamichhane, Aramva Bikram Adhikari

**Affiliations:** a Department of Medicine, Patan Academy of Health Sciences, Lalitpur, Nepal; b Department of Medicine, Nepal Medical College Teaching Hospital, Kathmandu, Nepal; c Department of Medicine, V N Karazin Kharkiv National University Kharkiv, Kharkiv, Ukraine; d Department of Medicine, Tribhuvan University Teaching Hospital, Institute of Medicine, Maharajgunj, Nepal

**Keywords:** adverse effects, anaphylaxis, case report, pantoprazole, PPI

## Abstract

**Introduction:**

Pantoprazole is a commonly used proton pump inhibitor (PPI) with rare incidents of dreaded and life-threatening complications.

**Case presentation:**

A 57-year-old woman presented to the emergency department with an acute onset of generalized rashes and chest tightness after intake of an over-the-counter oral pantoprazole and domperidone combination tablet. She later presented again with a headache for which she was administered intravenous acetaminophen and pantoprazole, following which she developed severe itching, hives, and shortness of breath with hypotension. She was managed with 0.5 ml of adrenaline (1:1000) intramuscularly and supportive measures after which she was stabilized and discharged.

**Discussion:**

Drug reactions and treatment history to oral pantoprazole are poorly recorded and explored due to large over-the-counter sales, and thus can be missed. Physicians should be aware of cross-hypersensitivity among PPIs, and a safe alternative should be decided after a skin allergy workup.

**Conclusion:**

It is necessary to extensively extract drug and allergy history, and even the most commonly used drugs with rarely any side effects should also be used cautiously.

## Introduction

Pantoprazole, a proton pump inhibitor (PPI) used in dyspepsia, peptic ulcer disease, and gastroesophageal reflux disease, is a safe and effective drug with rare incidents of serious adverse effects^[1,2]^. It exerts its action by irreversibly inhibiting the H+/K+ ATP pump in the parietal cells of the stomach, leading to decreased gastric acid production^[3]^. In a study conducted by Tucker in 1994, diarrhea, dizziness, nausea, and pruritus were the common side effects of pantoprazole^[4]^. Side effects of long-term use of pantoprazole include small intestinal bacterial overgrowth, magnesium deficiency, calcium deficiency, iron deficiency, vitamin B12 deficiency, bone demineralization, and interstitial nephritis^[5]^. Fortunately, dreaded and life-threatening complications like anaphylaxis, drug rash with eosinophilia, DRESS, toxic epidermal necrolysis, and Stevens–Johnson syndrome are among the rarest side effects of pantoprazole^[1]^.

**HIGHLIGHTS**

- Pantoprazole is a commonly used PPI with rare incidents of dreaded and life-threatening complications.
- A 57-year-old woman developed anaphylaxis after administration of IV pantoprazole.
- Drug reactions and treatment history to oral pantoprazole are poorly recorded.
- Safe alternative should be decided after a skin allergy workup.

This case report has been reported in line with the SCARE 2025 criteria^[6]^.

## Case history

A 57-year-old woman presented to the emergency department with an acute onset of generalized rashes and chest tightness after intake of an over-the-counter oral pantoprazole and domperidone combination tablet 1 h back. Her blood pressure (BP) was 110/70, pulse was 120 bpm, and SpO_2_ was 97% in room air at presentation. Her hemogram, random blood sugar, renal function tests, and liver function tests were within normal limits.

She was given 45.5 mg of intravenous (IV) pheniramine, 100 mg of IV hydrocortisone with 50 mg of IV ranitidine, and nebulization done with salbutamol and ipratropium, after which her symptoms subsided. She was admitted for observation, which was uneventful, and was discharged the following day.

Eighteen days later, the same patient presented to the emergency department with a headache for 1 day, generalized, with no aggravating or relieving factors, and no associated symptoms. She had initially taken 500 mg of acetaminophen tablet, which did not relieve the headache. Her vitals were stable (BP of 140/90 mmHg, pulse 83 bpm, and SpO_2_ 98% at room air) at presentation, and her blood hemogram, renal function test, and urine routine microscopic examination were within normal limits.

She was administered 40 mg of IV pantoprazole, followed by 1 g of IV acetaminophen. Immediately after receiving the medications, she complained of severe itching without any visible rashes, although her vitals remained stable. She was given 45.5 mg of IV pheniramine, which did not improve her condition, and she developed hives around her abdomen and bilateral upper limbs. Then she received 100 mg of IV hydrocortisone injection. A minute later, despite the administration of these medications, she reported shortness of breath, nausea, and chest discomfort. At that time, her BP was 80/50 mmHg, oxygen saturation was 73% on room air, and her pulse was 122 bpm with weak pulsation. She appeared anxious, nauseated, and was sweating. Immediately thereafter, she received 0.5 ml of adrenaline (1:1000) intramuscularly, along with oxygen via face mask, IV normal saline, and 4 mg of ondansetron injection. Her condition improved, with BP stabilizing at 100/70 mmHg, saturation at 98% in room air, and pulse at 102 bpm. The vitals were recorded and documented in chronological order (Table 1). The patient was monitored for 24 h to observe for any recurrent symptoms and was subsequently discharged with instructions to undergo a drug allergy test. Desensitization was suggested but refused by the patient as it is not a routinely practiced procedure in a resource-poor setting such as our center.

**Table 1:** Comparison of vitals throughout the management

	On presentation	After onset of symptoms	After management
BP	140/90 mmHg	80/50 mmHg	100/70 mmHg
SpO_2_	98% in room air	73% in room air	98% in room air
Pulse	83 bpm	122 bpm	102 bpm

**Figure 1.**
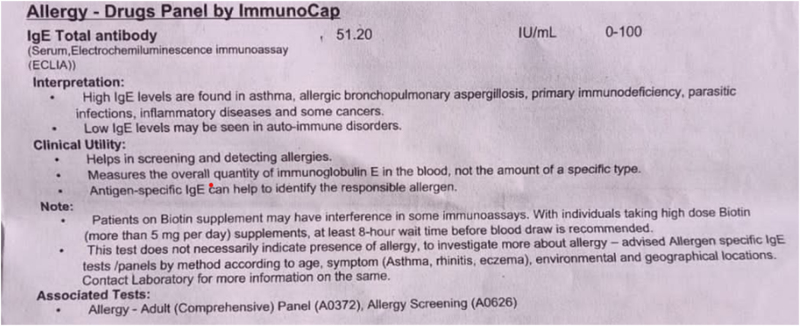
Total IgE level of the patient.

She underwent allergy tests for drug panel by serum enzyme immunoassay for commonly used drugs, which reported nonallergic to ciprofloxacin, human insulin, amoxicillin, ampicillin, penicilloyl G, penicilloyl V, ibuprofen, diclofenac, and acetaminophen, which ruled out acetaminophen allergy and pointed toward pantoprazole allergy. Her immunoglobulin E (IgE) level was found to be 51.20 IU/ml, which was within normal limits. (Fig. 1)

## Discussion

Drug reactions and treatment history to oral pantoprazole are poorly recorded and explored due to large over-the-counter sales, and thus can be missed. Only a few cases of anaphylactic shock and repeated occurrences of adverse events in the same patient to pantoprazole have been reported in the literature. In this article, we present the case of a 57-year-old woman with an adverse reaction to oral and IV pantoprazole administration. In this case report, we can observe the same 57-year-old patient developing an adverse reaction to both oral pantoprazole–domperidone combination and IV administration of pantoprazole, with a temporal relationship between drug administration and onset of symptoms. The second reaction with IV pantoprazole alone strengthens the association The patient was managed in accordance to the WAO 2020 guidelines^[7]^.

IgE-mediated anaphylaxis is the most frequent mechanism of anaphylaxis, which involves allergen-specific IgE interaction on mast cell while non-IgE-mediated anaphylaxis involves immunologic and nonimmunologic pathways like complement activation or IgG-mediated anaphylaxis^[7]^. IgE-mediated anaphylaxis was tested using serum enzyme immunoassay technique from patient’s blood samples. In a case of pantoprazole anaphylaxis during peribulbar anesthesia, it is highlighted that a bias to common agents like antibiotics could sometimes hinder the identification of root cause of anaphylaxis and might lead to further occurrence and thus requires vigilance and high suspicion of the clinician toward PPIs as identifying the cause is the main step in preventing such episodes in the future^[8]^. Physicians should also be aware of cross-hypersensitivity among PPIs, and a safe alternative PPI can usually be decided after a skin allergy workup^[9]^.

Kadoke *et al* reported a case of multiple adverse events 3 times in a 38-year-old patient with oral and IV administration of pantoprazole, which further makes us realize the importance of previous allergy history with due diligence to prevent such adverse events^[10]^. Although very rare, a case of pantoprazole-induced generalized tonic-clonic seizure has been reported by Anandan *et al*, attributed to hypomagnesemia in a patient with long-term use of pantoprazole^[11]^. Gázquez *et al* described a case Kounis syndrome in a 68-year-old man associated with PPI. It is an acute coronary syndrome characterized by functional and metabolic changes occurring in the heart in response to a serious allergic insult occurring through the arachidonic pathway^[12]^. If there is a need for the use of PPI despite cross-hypersensitivity, such as in Barrett’s esophagus, a desensitization protocol can be done under the supervision of doctors, nurses, and ready-to-use resuscitation equipment^[9]^.

In our patient, serum enzyme immunoassay IgE levels were normal, which left room for further investigation into the possible causes of anaphylaxis.

## Conclusion

Therefore, it is necessary to extensively extract drug and allergy history, and even the most commonly used drugs with rarely any side effects should also be used cautiously. This article highlights the need for doctors to inform and aware the patient about his/her drug allergy and vice versa.

## Data Availability

Not applicable.
